# Targeting cancers through TCR-peptide/MHC interactions

**DOI:** 10.1186/s13045-019-0812-8

**Published:** 2019-12-18

**Authors:** Qinghua He, Xianhan Jiang, Xinke Zhou, Jinsheng Weng

**Affiliations:** 1 Department of Center Laboratory, The Fifth Affiliated Hospital of Guangzhou Medical University, 621 Gangwan Rd, Huangpu Qu, Guangzhou, 510700 China; 2 Department of General Surgery, The Fifth Affiliated Hospital of Guangzhou Medical University, Guangzhou, 510700 China; 3 Department of Lymphoma and Myeloma, Division of Cancer Medicine, The University of Texas MD Anderson Cancer Center, 1414 Holcombe Boulevard, Houston, TX 77030 USA

**Keywords:** T cell receptor, Tumor antigen, Immunotherapy, Peptide

## Abstract

Adoptive T cell therapy has achieved dramatic success in a clinic, and the Food and Drug Administration approved two chimeric antigen receptor-engineered T cell (CAR-T) therapies that target hematological cancers in 2018. A significant issue faced by CAR-T therapies is the lack of tumor-specific biomarkers on the surfaces of solid tumor cells, which hampers the application of CAR-T therapies to solid tumors. Intracellular tumor-related antigens can be presented as peptides in the major histocompatibility complex (MHC) on the cell surface, which interact with the T cell receptors (TCR) on antigen-specific T cells to stimulate an anti-tumor response. Multiple immunotherapy strategies have been developed to eradicate tumor cells through targeting the TCR-peptide/MHC interactions. Here, we summarize the current status of TCR-based immunotherapy strategies, with particular focus on the TCR structure, activated signaling pathways, the effects and toxicity associated with TCR-based therapies in clinical trials, preclinical studies examining immune-mobilizing monoclonal TCRs against cancer (ImmTACs), and TCR-fusion molecules. We propose several TCR-based therapeutic strategies to achieve optimal clinical responses without the induction of autoimmune diseases.

## Introduction

Adoptive T cell therapy (ACT) strategies have achieved significant success in the past several years, as demonstrated by the recent approval of two chimeric antigen receptor-engineered T cell (CAR-T) therapeutic medicines by the Food and Drug Administration (FDA). Kymriah™ (tisagenlecleucel), the anti-cluster of differentiation 19 (CD19) CAR-T therapy produced by Novartis, has been approved for the treatment of pediatric patients and young adults with refractory or relapsed (R/R) B cell precursor acute lymphoblastic leukemia (ALL) [1]. Yescarta™ (axicabtagene ciloleucel), another anti-CD19 CAR-T therapy, produced by Kite’s company, was approved to treat adult patients with R/R large B cell lymphoma [2, 3]. The recent approval of these treatments has confirmed the dramatic effects of adoptive T cell therapy for the field of cancer therapy. Currently, multiple CAR-T therapeutic clinical trials are being performed, targeting various hematological cancer antigens, and some have demonstrated great anti-tumor effects [4]. However, CAR-T therapy against solid tumors has achieved limited success in clinical trials because few tumor-specific biomarkers are expressed on the surfaces of solid tumor cells [5–10].

Because cell membrane proteins constitute less than 15% of the whole cell protein population, and 85% of cellular proteins are intracellular, immunotherapies that target intracellular proteins have much greater application potential than therapies that target proteins on the cell membrane [11]. In 1974, Doherty and Zinkernagel discovered that fragments of foreign peptides on major histocompatibility complex (MHC) molecules can activate T cells of the same MHC alleles, providing the basic mechanism through which immune cells can recognize intracellular proteins via T cell receptor (TCR)-peptide/MHC interactions [12]. The subsequent cloning of the TCR α and β chains that specifically recognize the peptide/MHC have confirmed the existence of this molecular mechanism in the human body [13, 14]. In this model, intracellular proteins in human cells are digested by the proteasome digestion to become short peptides, which enter the endoplasmic reticulum (ER) and are conjugated with the MHC molecule for presentation on the cell surface [15]. These peptide/MHCs can be recognized by autologous or allogeneic T cells that contain the same MHC alleles through TCR-peptide/MHC interactions [16]. T cells can exert specific immune surveillance functions, by secreting cytotoxic granules, cytokines, or perforin to mediate cell apoptosis. In addition, most tumor-specific antigens that control cell growth, proliferation, and death are intracellular; therefore, this pathway has been widely explored to eliminate tumor- and virus-infected cells [17, 18]. Numerous studies have demonstrated the feasibility of eliminating tumor cells via tumor antigen-specific T cells by targeting the TCR-peptide/MHC interaction on the tumor cell surface [19–21].

The early studies examining the TCR-peptide/MHC interaction used only a small number of T cells that were cultured in a laboratory environment, and the process required to generate tumor antigen-specific T cells is complicated and expensive. With advances in genetic engineering technologies, people have found that cloning the tumor antigen-specific TCRs and transducing the TCRs into normal T cells by lentivirus or retrovirus can quickly imbue normal T cells with antigen-specific recognition abilities [22]. These have brought the advancement of TCR-engineered T cell therapy (TCR-T). Currently, there are more than 84 TCR-T immunotherapy clinical trials registered on the clinictrials.gov website, indicating the great potential for TCR-T in cancer immunotherapy [23]. Here, we review the TCR constructs, TCR signaling pathways, and the effects and toxicity associated with TCR-T immunotherapy in clinical trials. We also discuss other TCR-based molecules, such as immune-mobilizing monoclonal TCRs against cancer (ImmTACs), TCR-fusion proteins, and TCR-multimer molecules. Finally, we compare the advantages and disadvantages of various TCR-based immunotherapies with other strategies.

## TCR constructs and signaling pathways

**Fig. 1:**
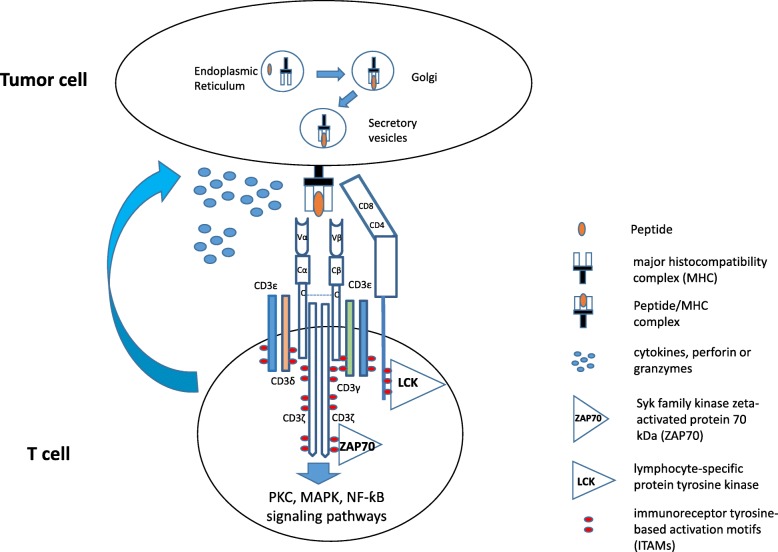
Schematics of TCR-peptide/MHC interactions. In human, 95% of T cells express a pair of TCR α and β chains with six CD3 chains (CD3γ, CD3δ, 2 CD3ε, and 2 CD3ζ) and CD8 or CD4 co-receptors on the cell surface. Each CD3 chain contains one to three ITAMs at the intracellular domain. After encountering the antigen-specific peptide/MHCs expressed on the surface of tumor cells, T cells activate ITAMs, ZAP70, PKC, MAPK, NF-κB signaling pathways, and secret perforin, granzymes, and cytokines, leading to the lysis of tumor cells. ITAMs, immunoreceptor tyrosine-based activation motifs; ZAP70, Syk family kinase zeta-activated protein 70 kDa; MAPK, mitogen-activated protein kinase; PKC, protein kinase C; NF-ƙB, nuclear factor kappa-light-chain-enhancer of activated B cells; LCK, lymphocyte-specific protein tyrosine kinase

The native TCRs on T cells consist of four distinct T cell antigen receptor polypeptides (α, β, γ, and δ) that form two different heterodimers (α:β and γ:δ). Approximately 95% of T cells in the peripheral blood consist of α:β chains and 5% of peripheral blood T cells consist of γ:δ chains [24]. In the human genome, the T cell receptor α chain (TCRA) contains at least 50 functional T cell receptor α chain variable (TRAV) gene segments, and the T cell receptor β chain (TCRB) is known to contain at least 75 functional T cell receptor β chain variable (TRBV) gene segments, which combine to form approximately 10^15^–10^21^ different TCRs in the human body [25, 26]. TCRs have very short intracellular domains; therefore, their signaling pathways depend heavily on the CD3 protein complex (CD3ζ, CD3δ, CD3ε, and CD3γ), CD8, and CD4, which act as co-receptors that are located in close proximity to TCRs [27]. Each CD3 chain contains one to three immunoreceptor tyrosine-based activation motifs (ITAMs) in the intracellular domain (Fig. 1). After engaging with antigen-specific peptide/MHCs, TCRs are thought to trigger a conformational change in the TCR-CD3 complex that activates the Src kinases leukocyte-specific tyrosine kinase (LCK) and Fyn to phosphorylate ITAMs [28]. Phosphorylated ITAMs then recruit and activate the Syk family kinase zeta-activated protein 70 kDa (ZAP70), which phosphorylates other proteins, such as the trans-membrane linker for activation of T cells (LAT), leukocyte protein of 76 kDa (Slp-76), and interleukin-2 inducible tyrosine kinase (ITK) [29]. These activated molecules then form a signalosome scaffold to activate the protein kinase C (PKC), mitogen-activated protein kinase (MAPK), and nuclear factor kappa-light-chain-enhancer of activated B cells (NF-κB) signaling pathways in T cells, leading to cytokine secretion, granule secretion, cell movement, and cell proliferation [30]. Thus, the binding of TCRs with the peptide/MHC represents the most important step for T cell activation, differentiation, and proliferation.

## Pre-clinical studies of TCR-T therapy

In 1986, DembiĆ and colleagues first isolated the TCR α and β chains that specifically recognized the hapten fluorescein (FL) on the mouse MHC class I Dd allele from the (C57BL/6 × DBA/2) F1 mouse cytotoxic T cell clone BDFL 1.1.3 (called BDFL) [31]. Using the protoplast fusion method, they transferred the 31 genetic BDFL alleles into another T cell and found that the expression of the TCR α and β genes endowed the recipient cells with the specificity of the donor cells. This early study used whole genomic DNA fragments during the transfection, and the efficiency was very low. Nevertheless, they demonstrated the feasibility of cloning and transferring an antigen-specific TCR from one T cell to another T cell to generate antigen specificity. In a later study, Kessels transduced a mouse MHC class-I-restricted TCR targeting an influenza virus epitope into mouse T cells by retroviral infection. They found that the genetically modified T cells could be activated by the specific virus antigen in vivo, was home to effector sites, and contributed to tumor clearance. The T cell clone expanded greatly after in vivo antigen encounter and completely eliminated virus epitope-expressing, syngeneic EL4NP thymoma cells after four days of incubation. Even though the transgenic TCRs were specific for viral antigens, rather than for true tumor antigens, these in vivo results provided solid evidence that the adoptive transfer of TCR-engineered T cells could potentially eliminate tumor cells in vivo [32].

Since then, many TCRs that target peptide/MHCs derived from tumor- or virus-associated/specific antigens have been cloned and expressed in normal T cells, to redirect T cell specificity, including TCRs targeting the following: an epitope derived from melanoma-associated antigen 3 (MAGE-A3) [33]; melanoma antigen recognized by T cells 1 (MART-1) [34–36]; human immunodeficiency virus (HIV) Gag and Pol antigens [37, 38]; hepatitis C virus (HCV) non-structure protein 3 (NS3) [39]; Epstein-Barr virus (EBV) [40]; latent membrane protein 2 (LMP2) [41]; mouse double-minute 2 (MDM2) [42]; New York esophageal squamous cell carcinoma-1 (NY-ESO-1) [43]; melanoma-associated antigen 1 (MAGE-A1) [44]; glycoprotein 100 (gp100) [45, 46]; tumor protein p53 (P53) [47]; human papillomavirus (HPV) 16E7 [48]; minor histocompatibility antigens (mHag) [49]; minor histocompatibility antigen HA-1 (HA-1) [50]; ubiquitously transcribed tetratricopeptide repeat gene on the Y chromosome (UTY) [51]; ribosomal protein S4, Y-linked (RPS4Y) [52]; tyrosinase [53]; the MHC class-II-restricted dead-box RNA helicase Y (DBY) [54]; cytotoxic T cell (CTL)-recognized antigen on melanoma (CAMEL) [55]; Wilms’ tumor 1 (WT1) [56, 57]; a renal cell carcinoma (RCC) tumor antigen [58]; mouse mastocytoma P815 [59]; and carcinoembryonic antigen (CEA) [60]. Pre-clinical studies of these TCRs have demonstrated that the TCR-transduced T cells can recognize tumor cells expressing the specific antigen with the same MHC alleles.

In these studies, the in vitro stimulation of peripheral blood mononuclear cells (PBMC) or tumor-infiltrating lymphocytes (TILs) from normal donors or patients was the primary method used to generate and clone tumor antigen-specific TCRs [57, 61]. TCRs that specifically recognize the peptide/MHC were then transduced into normal T cells isolated from donors or patients by retroviral or lentiviral methods [35]. Due to negative selection in the thymus, TCRs isolated from peripheral blood often have low affinity for cancer cells [62, 63]. However, thymus selection is not perfect, and high-affinity TCRs have been successfully isolated from peripheral blood [64, 65]. Another method for isolating tumor antigen-specific TCRs has been performed using human MHC allele-transgenic mice [47]. For this method, tumor antigens were emulsified with an adjunct and injected into MHC-transgenic mice. After several rounds of injections, the mouse spleen was removed, and tumor-specific TCRs were cloned and transduced into human PBMCs. The advantage of this method is that the mouse TCRs do not encounter any human antigens in the thymus and can have a high affinity for human antigens. Therefore, many TCRs have been isolated using this method, including TCRs targeting the peptide/MHCs for MDM2 [42], gp100 [66], CEA [60], and p53 [47]. However, mouse-derived TCRs are foreign to the human body, and immune responses against mouse TCRs have been observed in patients [67]. Another method for isolating tumor antigen-specific TCRs utilizes display technology [68–70]. In this method, a phage library that expresses human TCR α and β chains was mixed with tumor antigen-specific peptide/MHCs. After several rounds of selection, the TCR with the highest binding affinity for the peptide/MHC can be selected and used to genetically engineer T cells. One advantage of phage library-derived TCRs is that they can bind to peptide/MHCs with reduced stability. However, because of the lack of the thymus-selection process, the TCRs isolated from phage libraries can be damaging to normal tissues [71].

**Fig. 2:**
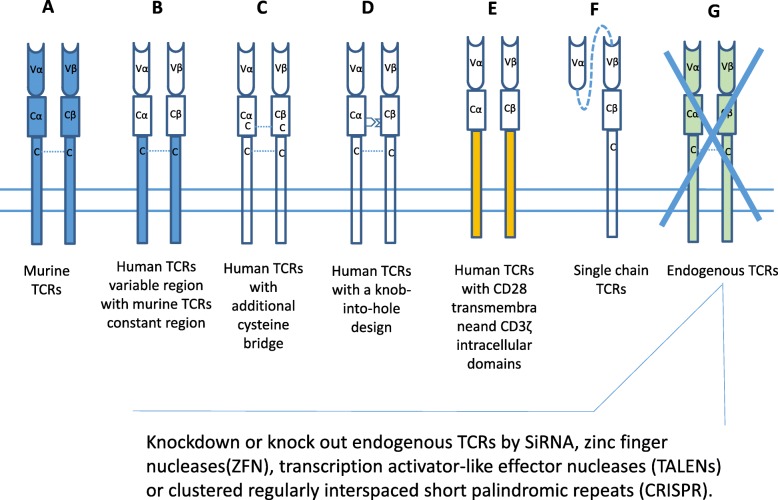
Schematics of the methods used to prevent the mismatch between transduced TCRs and endogenous TCRs. (**a**) TCRs derived from MHC-transgenic mice. (**b**) Human TCRs variable region chimerized with murine TCRs constant region. (**c**) Human TCRs with an additional cysteine bridge at TCRs constant region. (**d**) Human TCRs with a knob-into-hole design at TCRs constant region. (**e**) Human TCRs chimerized with CD28 transmembrane and CD3ζ intracellular domains. (**f**) Single-chain TCRs (scTCRs). (**g**) knockdown or knockout of endogenous TCRs by SiRNA, zinc finger nucleases (ZFN), transcription activator-like effector nucleases (TALENs), or by clustered regularly interspaced short palindromic repeats (CRISPR)

Recipient T cells also express endogenous TCR α and β chains, which could pair with the transduced tumor antigen-specific TCR α and β chains and cause harmful autoimmune diseases [72, 73]. To prevent this result, several strategies have been developed during preclinical studies. The first method replaced the constant region of the human TCR with a murine TCR constant region [74]. Because mouse TCR α and β chains have less capacity to pair with human TCR α and β chains, this method can reduce the mispairing of transferred TCR α and β chains with endogenous TCR α and β chains. Another method is to introduce mutations into the transferred TCR α and β chains, by generating an extra cysteine bridge into the constant region [75], mutating key amino acids found at the interfaces between constant regions [76], or convert the transferred TCR α and β chains into a single-chain TCR (scTCR) structure [77]. Genetically ligating the TCRs with the CD28 transmembrane domain and CD3ε can also reduce the mispairing of TCR α and β chains [78] (Fig. 2).

The deletion or silencing of the expression of endogenous TCR α and β chains in recipient T cells can also greatly reduce the mispairing between transduced TCR α and β chains with endogenous TCR α and β chains. The silencing of endogenous TCRs α and β chains can be achieved through the use of small-interfering RNAs (siRNA) [79, 80], zinc finger nucleases (ZFNs) [81, 82], transcription activator-like effector nucleases (TALENs )[83], or by clustered regularly interspaced short palindromic repeats (CRISPR) technology (Fig. 2) [84]. These approaches can additionally enhance TCR surface expression and effector function. Transferring TCR genes into hematopoietic stem cells (HSCs) or γδ T cells can also generate antigen-specific T cells, without the mispairing of TCR α and β chains [85, 86]. Although the TCR mispairing phenotype has not been observed in a clinic [87], the silencing of endogenous TCRs was shown to reduce the occurrence of the lethal graft versus host disease (GvHD) in a mouse model [88].

## Clinical studies of TCR-T immunotherapy

Tumor antigens are grouped into several categories in a clinic, according to their origins and specificity. The first category is oncovirus antigens, which include Epstein-Barr nuclear antigen 1–3 (EBNA 1–3), latent protein 1 (LMP1) and LMP2 derived from EBV [89], hepatitis B virus X protein (HBX) from hepatitis B virus (HBV) [90, 91], and type E5, E6, and E7 proteins from HPV [92]. The second group is neoantigens, which are derived from chromosomal and genetic mutations in tumor cells, which include beta-catenin S37F in melanoma [93], alpha-actinin-4 K122 N in lung cancer [94], and heat shock protein 70 kilodalton-2 (hsp70-2) F293I in renal cancer [95]. The third group of tumor antigens is the cancer-testis (CT) antigens, which are overexpressed in multiple types of tumor cells [96, 97], and in healthy donors, this group of antigens is expressed only in immune-privileged organs, such as the testis or placenta. The fourth group of tumor antigens involves antigens with minimal or limited expression in normal cells, such as MART-1, gp100, and tyrosinase [20, 98, 99]. Both oncovirus antigens and neoantigens are tumor-specific. However, viral infections cause only about 10–15% of all human cancers [100]. Neoantigens are patient-specific, with interpatient tumor heterogeneity, intratumor heterogeneity, and intermetastatic heterogeneity [101]. Moreover, the procedure for identifying genetic mutations and preparing TCR-based therapies for each patient is tedious and expensive [102], which has hampered the wide application of TCR-based cellular immunotherapies that target oncovirus antigens and neoantigens in a clinic. Currently, TCR-based immunotherapies in clinical trials primarily focus on tumor-associated antigens and CT antigens (Table 1). 
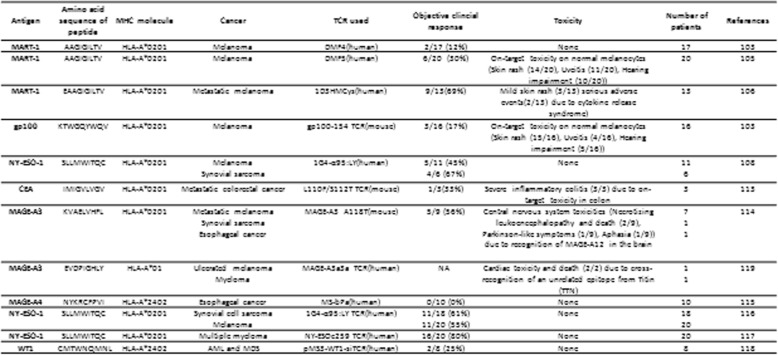

**Table 1:** Information of clinical trials of TCR-engineered T cells

Antigen	Amino acid sequence of peptide	MHC molecule	Cancer	TCR used	Objective clincial response	Toxicity	Number of patients	References
MART-1	AAGIGILTV	HLA-A*0201	Melanoma	DMF4(human)	2/17 (12%)	None	17	103
MART-1	AAGIGILTV	HLA-A*0201	Melanoma	DMF5(human)	6/20 (30%)	On-target toxicity on normal melanocytes (Skin rash (14/20), Uveitis (11/20), Hearing impairment (10/20))	20	105
MART-1	EAAGIGILTV	HLA-A*0201	Metastatic melanoma	1D3HMCys(human)	9/13(69%)	Mild skin rash (3/13) serious adverse events(2/13) due to cytokine release syndrome	13	106
gp100	KTWGQYWQV	HLA-A*0201	Melanoma	gp100-154 (mouse)	3/16 (17%)	On-target toxicity on normal melanocytes (Skin rash (15/16), Uveitis (4/16), Hearing impairment (5/16))	16	103
NY-ESO-1	SLLMWITQC	HLA-A*0201	Melanoma	1G4-α95:LY(human)	5/11 (45%)	None	11	108
			Synovial sarcoma		4/6 (67%)		6	
CEA	IMIGVLVGV	HLA-A*0201	Metastatic colorectal cancer	L110F/S112T (mouse)	1/3(33%)	Severe inflammatory colitis (3/3) due to on-target toxicity in colon	3	113
MAGE-A3	KVAELVHFL	HLA-A*0201	Metastatic melanoma	MAGE-A3 A118T(mouse)	5/9 (56%)	Central nervous system toxicities (Necrotizing leukoencephalopathy and death (2/9), Parkinson-like symptoms (1/9), Aphasia (1/9)) due to recognition of MAGE-A12 in the brain	7	114
			Synovial sarcoma				1	
			Esophageal cancer				1	
MAGE-A3	EVDPIGHLY	HLA-A*01	Ulcerated melanoma	MAGE-A3a3a (human)	NA	Cardiac toxicity and death (2/2) due to cross-recognition of an unrelated epitope from Titin (TTN)	1	119
			Myeloma				1	
MAGE-A4	NYKRCFPVI	HLA-A*2402	Esophageal cancer	MS-bPa(human)	0/10 (0%)	None	10	115
NY-ESO-1	SLLMWITQC	HLA-A*0201	Synovial cell sarcoma	1G4-α95:LY (human)	11/18 (61%)	None	18	116
			Melanoma		11/20 (55%)		20	
NY-ESO-1	SLLMWITQC	HLA-A*0201	Multiple myeloma	NY-ESOc259 (human)	16/20 (80%)	None	20	117
WT1	CMTWNQMNL	HLA-A*2402	AML and MDS	pMS3-WT1-siTCR(human)	2/8 (25%)	None	8	118

**Fig. 3:**
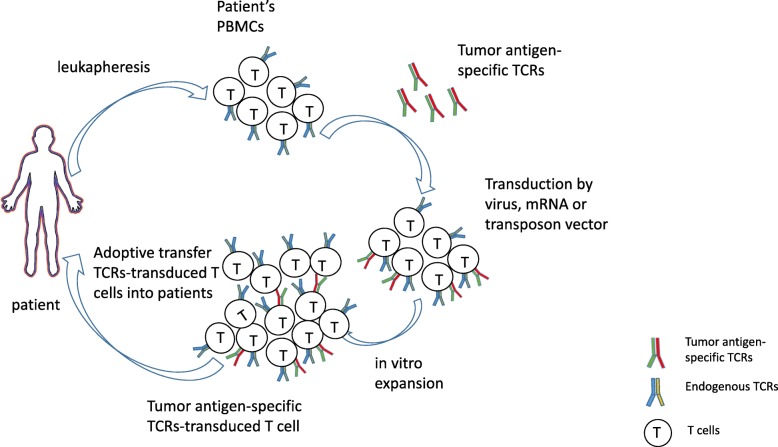
Schematics of TCR-T immunotherapy in current clinical settings. Peripheral blood mononuclear cell (PBMC) were isolated from the cancer patients by leukapheresis and transduced with tumor antigen-specific TCR-containing lentivirus, retrovirus, mRNA, or transposon vector. The tumor antigen-specific TCRs-transduced T cells were then expanded in vitro to a great number before infusion back into the patients

Morgan et al. reported the first TCR-T immunotherapy against melanoma in 2006 [103]. Using the RNA electroporation method, they transduced four RNAs, encoding TCRs that recognized MART-1:27–35, gp100:209–217, NY-ESO-1:157–165, and p53:264–272 peptide/human leukocyte antigen (HLA) A2, into the PBMCs of patients (Fig. 3). All of the transduced PBMCs were able to express the TCRs and specifically recognized peptide-pulsed T2 cells and antigen-expressing/HLA A2+ tumor cells through cytokine secretion. The MART-1 specific TCR (DMF4), which targeted the HLA A2-restricted AAGIGILTV peptide, was used in 17 melanoma patients, and more than 10% of peripheral lymphocytes from patients expressed the MART-1-specific TCRs for at least 2 months after the infusion. Of the 17 enrolled patients, who are all resistant to current therapies for metastatic diseases, two patients demonstrated the sustained objective regression of their metastatic melanomas, as assessed by the standard response evaluation criteria in solid tumors (RECIST) [104]. One patient, after treatment with the ACT protocol described above, experienced the complete regression of the axillary mass and an 89% reduction of the liver mass. He remains clinically disease-free, 21 months after treatment. Another patient experienced a regression of the hilar mass that measured 4.0 × 2.5 cm in the lungs and remained clinically disease-free for 20 months after treatment. A similar phenomenon has been observed during later clinical trials using MART-1-specific TCR-T immunotherapy. In 2009, Johnson et al. reported the results of a clinical trial, using an affinity-enhanced MART-1-specific TCR (DMF5) that recognized the MART-1 AAGIGILTV peptide, in 20 patients with metastatic melanoma. Six of them (30%) experienced objective cancer regression, with tumor shrinkage in the lung, brain, liver, lymphoma nodes, subcutaneous site, and skin [105]. In 2014, Chodon et al. reported the results of another trial, using a MART-1-specific TCR that targeted the HLA A2-restricted EAAGIGILTV peptide, in 14 melanoma patients, with the addition of dendritic cells (DC) vaccine pulsed with the same peptide. They found that 9 of the 13 treated patients (69%) showed evidence of tumor regression in multiple organs. Two patients demonstrated a time course-dependent decrease in the sizes of lung metastases, as assessed by serial chest X-rays, and one patient experienced the regression of large subcutaneous/muscle metastases, as assessed by computed tomography scan images. The peripheral blood reconstitution of MART-1-specific T cells peaked within 2 weeks of ACT, indicating rapid in vivo expansion. This study indicated that ACT using TCR-engineered T cells, with a very short ex vivo manipulation period and DC vaccine, is feasible and resulted in anti-tumor activity [106].

In 2009, Johnson et al. reported the results of a clinical trial, using a TCR-T therapy that specifically targeted the HLA A2-restricted gp100 antigen KTWGQYWQV in melanoma in 2009 [105, 107]. The gp100:154-162 epitope from the gp100 melanoma-melanocyte antigen is the most highly expressed peptide from this protein and is displayed on the cell surface. Attempts to generate a high-avidity human TCR against this epitope have been unsuccessful. Thus, they used a highly avid TCR that was generated in HLA A2 transgenic mice, and they found that 3 out of 16 (17%) patients experienced objective clinical responses after receiving the gp100-specific TCR-T cells [105], with metastatic tumors regressing in multiple organs, including the brain, lung, liver, lymph nodes, and subcutaneous sites.

Robbins et al. reported the first clinical trial results for TCR-T immunotherapy targeting NY-ESO-1 in synovial cell sarcoma and melanoma patients in 2011 [108]. The NY-ESO-1 antigen is a member of the CT gene family and is expressed in 15–50% of highly prevalent tumors, including breast, lung, prostate, and ovarian cancers [109]. As many as 60% of advanced myelomas have been reported to express NY-ESO-1, which correlated with tumor proliferation and high-risk features [110, 111]. Among advanced synovial cell sarcoma patients, 80% were found to express NY-ESO-1 [112]. In the study, they performed ACT with genetically engineered cells that targeted the NY-ESO-1 SLLMWITQC peptide/HLA A2 and found objective clinical responses in four of six (67%) patients with synovial cell sarcoma and five of 11 (45%) patients with melanoma bearing tumors expressing NY-ESO-1. Two out of 11 patients with melanoma demonstrated complete regressions that persisted after 1 year. A partial response, lasting 18 months, was observed in one patient with synovial cell sarcoma. These observations indicated that TCR-based gene therapies directed against NY-ESO-1 represent a new and effective therapeutic approach for patients with melanoma and synovial cell sarcoma. This trial represented the first successful treatment of nonmelanoma tumors using TCR-transduced T cells.

Parkhurst et al. reported the first clinical trial results using a TCR-T therapy targeting CEA in colon cancer patients in 2011 [113]. CEA is a glycosylated protein that is overexpressed in multiple gastrointestinal cancer cells. Three patients with metastatic colorectal cancer, who were refractory to standard treatments, received autologous T lymphocytes that were genetically engineered to express a murine TCR against the CEA IMIGVLVGV peptide/HLA A2. Profound decreases in serum CEA levels (74–99%) were detected in all three patients, and one patient experienced an objective regression of cancer metastatic to the lung and liver.

In 2013, Morgan et al. reported the results of a clinical trial using a TCR-T therapy targeting MAGE-A3 KVAELVHFL, which is an HLA A2-restricted epitope in synovial sarcoma, esophageal cancer, and metastatic melanoma patients. Five out of nine patients experienced the clinical regression of their cancers, based on the RECIST. Two patients experienced continued responses [114]. Patients who had metastatic melanoma in the lung, subcutaneous and intra-abdominal sites, mesenteric lymph nodes, or rib demonstrated an up to 89% decrease in the tumor size, which lasted from 4 to more than 15 months following treatment.

Kageyama et al. reported the clinical trial results of a TCR-T therapy targeting the HLA A2402-restricted MAGE-A4 epitope NYKRCFPVI in 10 patients with recurrent esophageal cancer in 2015. The patients were given sequential MAGE-A4 peptide vaccinations following the TCR-T therapy [115]. None of the patients exhibited tumor shrinkage in the short term, and all patients exhibited tumor progression within 2 months after the treatment. However, three patients who had minimal disease at the time of cell transfer remained free from disease progression for more than a year, without any further treatment.

Robbins et al. reported the results of a clinical trial using an affinity-enhanced TCR that recognized the NY-ESO-1 SLLMWITQC/HLA A2 epitope in 2015. They retrovirally transduced the TCR into PBMCs from 18 patients with synovial cell sarcomas and 20 patients with melanomas, who were resistant to current treatments. Eleven of 18 patients with NY-ESO-1(+) synovial cell sarcomas (61%) and 11 of 20 patients with NY-ESO-1(+) melanomas (55%) who received NY-ESO-1-specific TCR-T cells demonstrated objective clinical responses [116]. In the same year, Rapoport et al. reported the results of another clinical trial using a TCR that targeted the HLA A2-restricted NY-ESO-1 and LAGE-1 shared epitope SLLMWITQC in 20 myeloma patients. They used lentiviral transduction technology to engineer the T cells, and 20 patients with antigen-positive multiple myeloma (MM) received an average of 2.4 × 10^9^ engineered T cells 2 days after autologous stem cell transplant. They observed that 14 of the 20 (70%) patients experienced either a near-complete response (nCR, defined as a myeloma monoclonal band detectable only by sensitive immunofixation assay) or a CR, 2 patients had a very good partial response (VGPR; ≥ 90% reduction in paraprotein levels), 2 had a partial response (50–90% reduction), 1 had stable disease (< 50% reduction), and 1 had progressive disease. An overall 80% encouraging clinical response rate was observed for this trial [117].

In 2017, Tawara et al. reported the first clinical trial study using a WT1-specific TCR-T therapy [118]. WT1 is a tumor-associated antigen that is expressed constantly in leukemic cells during acute leukemia and myelodysplastic syndrome (MDS). Eight patients with refractory acute myeloblastic leukemia (AML) and high-risk MDS received two doses of 2 × 10^8^ WT-1-specific TCR-T cells, at a 4-week interval, associated with a mutated WT1 CYTWNQMNL peptide vaccine. Two patients showed transient decreases in blast counts in bone marrow, which was associated with hematopoiesis recovery. Four out of five patients who had persistent T cells at the end of the study survived longer than 12 months. For those who did not have persistent T cells in the peripheral blood, only one patient survived longer than 12 months.

## The toxicity of TCR-T immunotherapy

Although TCR-T immunotherapy has been shown to have dramatic anti-tumor effects in clinical trials, their toxicity is also very obvious. Of the clinical trials mentioned above, most were associated with some adverse effects, ranging from a mild skin rash to the severe death of patients, depending on the antigen targeted, the affinity of the TCR used, and the methods used to engineer the T cells (Table 1).

In the MART-1-specific TCR-T clinical trial reported by Morgan et al. in 2006, no specific toxicity has been identified in the two positively responding patients, despite expressing high levels of circulating MART-1-specific gene-transduced T cells in their bodies for longer than 1 year (between 20 and 70%) [103]. In the study reported by Johnson et al. in 2009, 29 of the 36 (80%) patients exhibited a widespread erythematous skin rash, with prominent epidermal spongiosis, necrotic epidermal keratinocytes, and a dense infiltrate of CD3+ T lymphocytes on biopsy. In addition, 14 of 20 DMF5 patients and 13 of 16 gp100 patients demonstrated the destruction of epidermal melanocytes, starting as early as day 5 after treatment. Local steroid administration, to treat uveitis and hearing loss, was required for these side effects [105]. In the trial reported by Chodon et al. in 2014, three patients who had evidence of transient tumor responses according to the results of serial X-rays and positron emission tomography (PET) scans also experienced a pronounced whole body erythematous skin rash. Two of them had serious adverse events (SAE) of acute respiratory distress requiring intubation associated with patchy pulmonary infiltrates within 1 week of cell infusion, resulting in the discontinuation of this cohort due to increased toxicities. Analyses of plasma from the peripheral blood indicated the production of multiple cytokines and the development of a cytokine storm. Corticosteroid therapy was administrated to the two patients who recovered their baseline respiratory functions within 2 weeks [106].

In the CEA TCR-T clinical trial, grade 2 diarrhea was observed in patient 1 and grade 3 diarrhea was observed in patients 2 and 3. Diarrhea started on days 5–8 and persisted for approximately 2 weeks before slowly resolving to normal by 4–6 weeks. All three patients were febrile between days 7 and 9 and were hemodynamically stable but required fluid-replacement therapy. Sequential colonoscopies revealed the development of inflammatory colitis in all three patients. Immunohistochemical staining for CEA in these biopsies demonstrated the near-complete loss of CEA in the denuded colon specimens. Genetic and cellular analyses of biopsy samples, obtained from upper and lower endoscopies performed 6–11 days post-treatment, using polymerase chain reaction (PCR) and fluorescence-activated cell sorting (FACS) analyses indicated the presence of substantial numbers of the adoptively transferred lymphocytes in all patients.

In a MAGE-A3 TCR-T clinical trial reported by Morgan et al. in 2013, three out of nine patients experienced mental status changes, and two patients lapsed into comas and subsequently died, beginning 1–2 days post-infusion. Magnetic resonance imagining analyses of the two dead patients demonstrated periventricular leukomalacia, and autopsies of their brains revealed necrotizing leukoencephalopathy, with extensive white matter defects, associated with the infiltration of CD3(+)/CD8(+) T cells. Another patient developed Parkinson’s disease-like symptoms, which resolved over 4 weeks, and the patient fully recovered [114]. Immunohistochemical staining of the patient and normal brain samples demonstrated rare, positively-stained neurons using an antibody that recognizes multiple MAGE-A family members. The TCR used in this study recognized epitopes in MAGE-A3/A9/A12. Molecular assays performed on human brain samples, using real-time quantitative-PCR, nanostring quantitation, and deep-sequencing, indicated that MAGE-A12 was expressed in the human brain (and possibly MAGE-A1, MAGE-A8, and MAGE-A9).

In another MAGE-A3 TCR-T clinical trial, reported by Linette in 2013, an affinity-enhanced TCR-T that targeted the MAGE-A3 EVDPIGHLY epitope on the HLA A1 allele was used in myeloma and melanoma patients [119]. The first two treated patients developed cardiogenic shock and died within a few days of T cell infusion. Gross findings at autopsy revealed severe myocardial damage, and histopathological analysis revealed T cell infiltration. No MAGE-A3 expression was detected in heart autopsy tissues. The robust proliferation of the engineered T cells in vivo was documented in both patients. A beating cardiomyocyte culture, generated by induced pluripotent stem cell (iPSC) technology, triggered T cell killing, due to the recognition of an unrelated ESDPIVAQY peptide, derived from the striated muscle-specific protein titin [120].

Although serious toxicities have been identified during MART-1, CEA, and MAGE-A3 TCR-T clinical trials, as mentioned above, the clinical trials using NY-ESO-1, MAGE-A4, and WT1 TCR-T therapies have been quite safe. In the NY-ESO-1 clinical trial, reported by Robbins et al. [108], no toxicities were attributed to the transferred cells, although all patients experienced the transient neutropenia and thrombocytopenia induced by the preparative regimen and the transient toxicities associated with interleukin (IL)-2; however, all patients recovered after the completion of the treatment. In the trial reported by Kageyama et al. in 2015 [115], none of the 10 patients experienced any adverse events during the first 14 days after T cell transfer. In four patients, they observed skin reactions, such as redness and induration, graded as 1, at the peptide vaccine sites. In the NY-ESO-1 trial reported by Rapoport et al. [117], no treatment-related fatalities were reported, and all seven reported SAEs resolved. Seventeen adverse events occurred, which were likely associated with the treatment, all of which were scored as grade 3 or lower. Skin rash with lymphocytosis occurred in 3 out of 20 patients, and some patients experienced a diarrheal syndrome that occurred later than expected for melphalan-induced mucositis, which was confirmed to be autologous graft versus host disease (aGVHD) in three out of 20 patients. In the WT1 TCR-T clinical trial, no adverse events involving normal tissue were observed [118].

## Other types of immunotherapies targeting the TCR-peptide/MHC

**Fig. 4:**
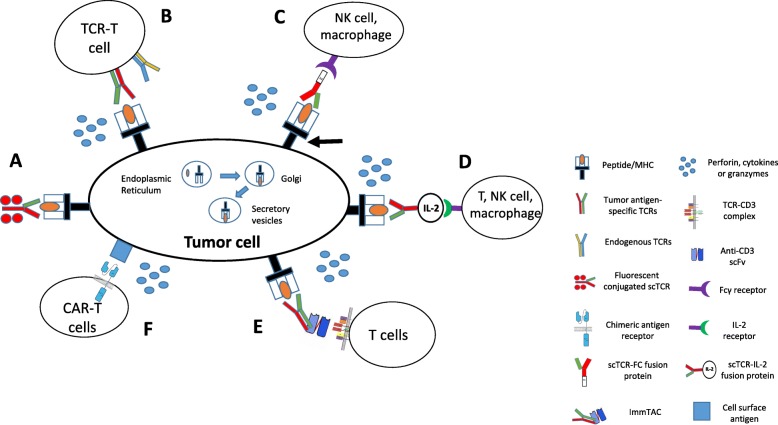
Schematics of the molecular mechanisms underlying TCR-based and CAR-T immunotherapy strategies. (**a**) Fluorescent-conjugated scTCRs. (**b**) TCR-T strategy. (**c**) scTCR-Fc fusion strategy. (**d**) scTCR-IL-2 fusion protein. (**e**) Immune mobilizing monoclonal TCRs against cancer (ImmTACs) strategy. (**f**) CAR-T strategy

Although TCR-T is the most common immunotherapy strategy targeting the TCR-peptide/MHC interaction, other TCR-based immunotherapy strategies have also been explored for clinical application. All of these strategies utilize a soluble TCR at one end, designed to recognize a specific peptide/MHC, and an immune cell activation motif [anti-CD3 single-chain fragment variable (scFv), IL-2 or fragment crystallizable (Fc)] at the other end, to activate the immune response (Fig. 4).

### ImmTAC

In 2012, Liddy et al. reported a new strategy for TCR-based immunotherapy that utilized a molecule named ImmTAC, or immune-mobilizing monoclonal TCRs against cancer [121]. In their study, four ImmTACs, each comprising a distinct tumor-associated antigen-specific monoclonal TCR with picomolar affinity targeting gp100, NYESO-1, MART-1, and MAGE-A3, were fused to a humanized anti-CD3 scFv, and expressed separately in the bacterial system, refolded and purified in vitro [122]. The formed dimers contained an anti-CD3 antibody at the end of TCR β chain, like bispecific T cell engagers (BiTEs), which could activate immune cells [123]. These ImmTAC molecules, when incubated with normal T cells at extremely low concentrations, effectively reprogramed T cells to kill melanoma cancer cells, both in vitro and in vivo, even when the cancer cells had extremely low surface epitope densities [121]. T cells in various memory compartments can be activated by ImmTAC molecules, and the induction of tumor cell lysis occurs in a serial manner. Later, this group extended their study to the colon, lung, myeloma, ovary, lymphoma, and bladder tumor models and found that the NY-ESO-1-specific ImmTAC was able to mediate the apoptosis of tumor cells, similar to melanoma cells [124]. The ImmTAC induced poly-functionality in both CD4 and CD8 T cells and potentiated antigen cross-presentation in dendritic cells [125, 126]. Two clinical trials (NCT01211262 and NCT02535078) have been initiated to test the effectiveness of these molecules [71].

### TCR-fusion proteins

In 2004, Card et al. reported the generation of a novel molecule (ALT-801, 264scTCR/IL-2), comprised of an anti-p53 (aa264–272) scTCR fused to an IL-2 molecule. The scTCR can specifically bind to tumor cell surfaces that express p53 peptide and the HLA A2 complex, and IL-2 can activate a broad range of immune cell types, including T cells, B cells, monocytes, macrophages, lymphokine-activated killer (LAK) cells, and natural killer (NK) cells, located in the proximity of tumor cells. They found that ALT-801 was able to mediate the specific killing of tumor cells in p53+/HLA-A2+ human melanoma (A375), breast cancer (MDA-MB231), and pancreatic carcinoma (PANC-1) xenograft models, in addition to having a fivefold longer terminal half-life than recombinant human IL-2 [127–129]. Based on these findings, ALT-801 was evaluated in a phase I study performed in patients with advanced malignancies. In the clinical trial, they found that 10 out of 26 patients showed stable disease for at least 11 weeks, while one complete response was observed in a patient with metastatic melanoma [130]. Another TCR-fusion molecule consisted of an scTCR specific for p53 (aa264–272) and the human immunoglobulin (Ig)G1 heavy chain constant region, including a Fc region to mediate antibody-dependent cell-mediated cytotoxicity (ADCC) [131]. This fusion protein (264scTCR/IgG1) was able to bind to an unmutated peptide derived from human p53 (aa 264–272) presented in the context of HLA-A2.1 and stimulate potent antitumor effects in a model of experimental non-small cell lung carcinoma (NSCLC) metastasis in nude mice through ADCC. A clinical phase I study for this molecule is planned for the treatment of p53+ NSCLC patients [132].

### scTCR/multimers

In addition to mediating cytotoxicity against tumor cells, the TCR-fusion protein can be used to directly visualize and quantify peptide/MHCs on unmanipulated human tumor cells [133]. In one study, the β constant region of scTCR was linked to a birA peptide tag to facilitate biotinylation and subsequent multimerization in the presence of streptavidin. This molecule was used to stain the peptide/MHCs on P53+/HLA A2+ tumor cells. They found that many tumor cells can be positively stained using this method. Tumor cells displaying as few as 500 peptide/MHC complexes were readily detectable by flow cytometry. The scTCR/multimers exhibited exquisite recognition capability and could distinguish peptides differing in as little as a single amino acid. Thus, scTCR/multimers represent a novel class of immunostaining reagents that can be used to validate, quantify, or monitor epitope presentation by cancer cells.

## Comparisons among TCR-based immunotherapy strategies and other immunotherapy strategies

Because the TCR α and β chains are membrane-bound proteins with hydrophobic properties [122], the transduction of TCRs into T cells represents the predominant form of TCR-based therapy. After transduction, the TCR α and β chains are able to pair with each other and to partner with CD3, CD4, and CD8 molecules expressed on the surface of T cells. Once the specific peptide/MHC is encountered, the TCRs can activate the CD3 complex to mediate an ITAM-dependent signaling pathway that lyses tumor cells [29, 30]. Because the intracellular domains of the CD3 complex contain multiple ITAMs to activate ZAP70, the signals of TCR-peptide/MHC interaction in T cells are amplified and it is reported that one copy of peptide/MHC complex can fully activate T cells to lyse tumor cells [134–136]. In addition, tumor antigen-specific TCR-T cells can persist for years in patients’ bodies. However, the in vitro preparation of TCRs for patient therapies can be time-consuming, without any guarantees for success. The TCR-T technique is complicated and costly and is associated with the risk of mispairing transduced TCRs with endogenous TCRs (Table 2). 
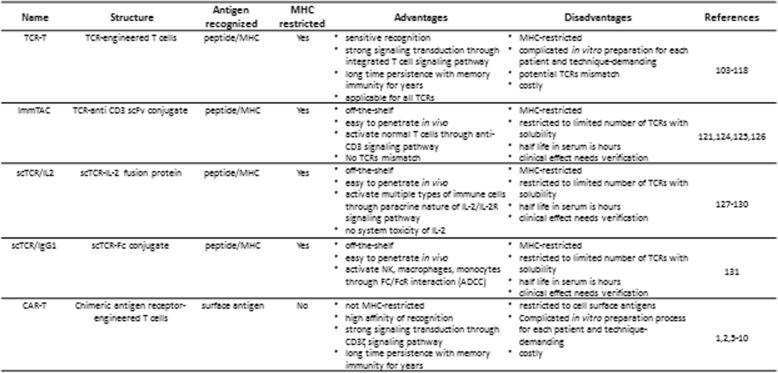

**Table 2:** Comparison of different TCR-based immunotherapy strategies with CAR-T therapy

Name	Structure	Antigen recognized	MHC restricted	Advantages	Disadvantages	References
TCR-T	TCR-engineered T cells	peptide/MHC	Yes	• sensitive recognition • strong signaling transduction through integrated T cell signaling pathway • long time persistence with memory immunity for years • applicable for all TCRs	• MHC-restricted • complicated in vitro preparation for each patient and technique-demanding • potential TCRs mismatch • costly	103-118
ImmTAC	TCR-anti CD3 scFv conjugate	peptide/MHC	Yes	• off-the-shelf • easy to penetrate in vivo • activate normal T cells through anti-CD3 signaling pathway • No TCRs mismatch	• MHC-restricted • restricted to limited number of TCRs with solubility • half life in serum is hours • clinical effect needs verification	121,124,125,126
scTCR/IL2	scTCR-IL-2 fusion protein	peptide/MHC	Yes	• off-the-shelf • easy to penetrate in vivo • activate multiple types of immune cells through paracrine nature of IL-2/IL-2R signaling pathway • no system toxicity of IL-2	• MHC-restricted • restricted to limited number of TCRs with solubility • half life in serum is hours • clinical effect needs verification	127-130
scTCR/IgG1	scTCR-Fc conjugate	peptide/MHC	Yes	• off-the-shelf • easy to penetrate in vivo • activate NK, macrophages, monocytes through FC/FcR interaction (ADCC)	• MHC-restricted • restricted to limited number of TCRs with solubility • half life in serum is hours • clinical effect needs verification	131
CAR-T	Chimeric antigen receptor-engineered T cells	surface antigen	No	• not MHC-restricted • high affinity of recognition • strong signaling transduction through CD3ζ signaling pathway • long time persistence with memory immunity for years	• restricted to cell surface antigens • Complicated in vitro preparation process for each patient and technique-demanding • costly	1,2,5-10

ImmTAC and TCR-fusion proteins are limited to those that have been successfully synthesized in vitro and can be fully dissolved in a solution. In vitro-synthesized TCRs tend to be low affinity because of a lack of association with CD3, CD4, and CD8 molecules; however, some genetic engineering can increase the affinity of in vitro-synthesized TCRs, as in ImmTACs [121, 137]. The advantages of in vitro-synthesized TCR-based therapy are that they do not need the in vitro preparation of a large number of tumor antigen-specific T cells and they are easy to penetrate the tissues and used as off-the-shelf. Moreover, they do not result in the mispairing of tumor antigen-specific TCRs with endogenous TCRs. However, their effect against cancers is waiting for more confirmation, as there are limited reports of ImmTAC or TCR-fusion proteins in clinical trials and their persistence in the serum is limited to several hours.

CAR-T therapy equips normal T cells with a tumor-cell-surface antigen-specific scFv that is ligated to the intracellular domain of CD3ζ. CAR-T therapy is not MHC-restricted but does require the in vitro preparation of antigen-specific T cells in large numbers. The affinities of the antibodies used in CAR-T therapy are generally higher than that for TCR; however, because of the lack of assistant CD4, CD8, or other CD3 molecules, the minimal concentration of antigen necessary to activate CAR-T cells is > 100 copies, and antigens with fewer copy numbers are unable to activate CAR-T cells [138, 139]. One drawback of CAR-T therapy is the lack of cell surface-specific biomarkers on solid tumor cells, which hampers the effects of CAR-T cells [5–10]. CAR-T therapies designed to target non-tumor-specific antigens on solid tumor cells resulted in severe toxicity in patients [8, 140].

## Strategies to overcome the toxicity of TCR-based immunotherapy

Tumor antigen-specific peptide/MHCs have been explored for many years as targets for therapeutic diagnosis and cancer immunotherapy. Numerous studies have demonstrated the feasibility of these strategies [19–21]. With solid evidence of tumor regression during clinical trials, we believe TCR-based immunotherapy represents an ideal target in our next step for cancer immunotherapy. However, significant toxicity has hampered the translation of TCR-T therapies into a clinic. Thus, methods for improving the safety and efficacy of TCR-T therapies are necessary. We propose the following strategies to further improve TCR-based therapies.

### First: the proper selection of TCR-targeted antigens

Based on the results from clinical trials, we found that TCR-T therapies that targeted tumor-associated antigens were generally associated with side effects or damage to normal tissues. MART-1 and gp100 are highly expressed in melanoma but are also expressed in normal melanocytes [141, 142], and CEA is expressed in normal colonic mucosa [99]. TCR-T targeting WT1 did not cause an autoimmune disease; however, the anti-tumor effect was also weak in this trial [118]. To avoid damaging normal tissues in future clinical trials, more sophisticated genetic engineering techniques are necessary, such as the titration of TCR affinity to only target tumor cells with high expression levels of the targeted peptide/MHC, without damaging normal tissues with low expression levels, or the development of double-specific T cells, as are used in CAR-T therapy [143, 144]. Alternatively, antigens from non-essential tissues can be targeted, such as CD19 and CD20 in B cells [145].

The CT family contains over 100 member proteins [146]. The first member of this family to be identified, MAGE-A1, was cloned by van der Bruggen and colleagues in 1991 [147]. The hallmark of this class of tumor-associated antigens is their restricted expression to germ-line tissues under normal conditions, whereas they are overexpressed in a variety of common epithelial malignancies, including cancers of the lung, breast, ovary, bladder, and melanoma [148]. The frequency of cancer-testis antigen (CTA) expression in these common cancers is generally in the range of 30–50% [112]. Due to their immunogenicity and frequency of expression, CTAs have been targeted during multiple cancer vaccine trials and ACT trials, using either CTL or TCR gene-modified T cells [149]. The function of CTAs remains largely unknown, although the MAGE-A family, containing 12 genes, has been suggested to function as adaptor proteins involved in transcriptional regulation, protein ubiquitination, and the regulation of the p53 pathway [150, 151]. The expression of CT genes has also been found to be associated with the development of malignant phenotypes and worse clinical outcomes [152, 153]. However, TCR-T therapy targeting CTA should be attempted cautiously, as demonstrated by the NY-ESO-1 and MAGE-A3 clinical trials [114, 117, 119]. Targeting NYESO-1 has been demonstrated to be relatively safe, but targeting MAGE-A3 was lethal for patients in two trials. These results indicate that each CTA member should be stringently screened to determine the extent of protein expression in human tissues. The rigorous bioinformatic screening of expression databases, such as IST/MediSapiens, Genevestigator, and BioGPS, which contain information from thousands of samples across a wide variety of healthy tissues, is also necessary. Even when the expression profile of a protein appears to represent an ideal target, the peptide sequence should be blasted using an in silico search (http://prosite.expasy.org/scanprosite/) to prevent the recognition of homologous peptides in other proteins. A peptide-scanning assay, with alanine or glycine replacement, should also be performed in the laboratory to exclude the recognition of degenerated peptides [120].

### Second: more complete safety screenings for TCR-based immunotherapy

Due to differences in protein sequences and expression profiles, mouse models are often considered to have little value when evaluating the safety of TCR-T therapies [154]. However, the toxicity observed in patients who received CEA-specific TCR-T therapy was highly similar to that observed in a CEA-transgenic model [155]. In this model, a CEA DNA vaccine was used to immunize wild-type mice, and CEA-specific T cells were collected from the spleen for ACT into CEA-transgenic mice. In addition to anti-tumor effects, the CEA-specific T cells damaged normal colon tissues, similar to autoimmune colitis, in the CEA-transgenic mice. In a premelanosome protein (Pmel-1) mouse model, ACT using gp100-specific T cells caused ocular damage, which paralleled the findings in human melanoma patients who received gp100-specific TCR-T therapy [156]. These findings indicate that mouse models with homologous human protein sequences and expression profiles can have value when performing safety screening for TCR-T therapies.

Human cell lines have been invaluable tools for scientists to screen for drug effect and safety. However, the interpretation of data from cell lines should be performed with caution. For example, in the MAGE-A3 trial, the initial screening of MAGE-A3 in formalin-fixed tissues revealed no MAGE-A3 expression in the heart. Co-culturing the TCR-T cells with primary cells derived from the heart also did not reveal any activity. In light of the obvious heart damage observed in two patients who died after MAGE-A3-specific TCR-T, researchers used a specific heart cell type, called icells, which are primary human heart cells immortalized by iPSC technology and can beat like normal heart cells under tissue culture conditions. Using this cell model, researchers found that MAGE-A3-specific TCR-T cells lysed the heart cells through the specific secretion of cytokines and cytotoxic granules [120]. Thus, the proper selection of primary cells that best reflect in vivo conditions is critical for TCR-T therapy safety screening.

### Third: methods to transduce the TCR into T cells, cell number, and phenotypes

In the trial reported by Morgan et al. in 2006, no significant toxicity was observed, partially because they used RNA electroporation instead of the stable transduction method [103, 157]. The transient expression of CARs or TCRs is safer than stable transduction during cell therapy [158, 159]. Moreover, the numbers and phenotypes of the transferred cells can also affect the toxicity. In the MAGE-A3 trial, patients who developed neurologic toxicity received a higher total number of cells, more CD3^+^/CD8^+^/Tetramer^+^ cells, and more T cells with a naïve phenotype [114]. This finding indicates that the modulation of the numbers and phenotypes of the transferred tumor antigen-specific TCR-T cells may affect the toxicity associated with TCR-T therapies. Recent studies reported the identification of a new subtype of T cells, called memory stem cells (T_SCM_), which can mediate dramatic anti-tumor effects at small numbers (4 × 10^6^), in vivo [160, 161]. T_SCM_ cells represent a clonally expanded primordial-memory subset, with increased proliferative and reconstitutive capacities. Moreover, several studies have demonstrated that CD4 T cells mediate better anti-tumor effects than CD8 T cells, by partnering with NK cells [162, 163]. T cells with potent anti-tumor effects have also been generated from TCR-transduced hematopoietic stem cells and induced pluripotent stem cells [22, 164, 165]. These studies have provided new tools for the engineering of T cells with tumor antigen-specific TCRs, although their effects require more thorough testing, both pre-clinically and clinically.

### Fourth: the optimization of generated TCR-T cell affinities

The avidity of a T cell, which is greatly dependent on the TCR affinity, has been shown to be directly correlated with its functions [166–168]. In the trial reported by Johnson et al. in 2009, they used a DMF5 TCR, which has a higher affinity than the DMF4 receptor to transduce the T cells, and they observed a higher response rate than that for the DMF4 trial [105]. High-affinity TCRs have been selected for most clinical trials because of their ability to recognize the peptide/MHCs at a low expression level on the surface of tumor cells. However, autoimmune diseases are frequently associated with high-affinity TCR-based therapies. Recently, several studies suggested that TCRs with low to medium affinities can mediate tumor destruction, without inducing autoimmune disease [144, 169–173]. Using seven gp100-specific TCRs, which spanned the physiological affinity range, Zhong and colleagues found that the TCR potency is determined by the TCR avidity, which reflects the combined contributions of both TCR affinity and CD8, rather than reflecting the TCR affinity alone. The killing of targeted cells, including the in vitro and in vivo lysis of tumor cells and autoimmunity, plateaued at an affinity threshold of approximately 10 μM, and TCRs with affinities higher than the 10-μM threshold did not lead to more potent anti-tumor activities [170]. The molecular mechanism underlying this effect is that maximal TCR clustering occurs at the 10-μM threshold, and further increases in the TCR affinity only lead to monovalent TCR-peptide/MHC interactions, which do not contribute to T cell functions. Furthermore, increasing TCR affinity can induce negative feedback mechanisms [174]. In the study by Miller et al. in 2019, they adoptively transferred CD8+ T lymphocytes expressing either a high-affinity or a low-affinity ovalbumin (OVA)-specific TCR into a RIP-mOVA mouse model, expressing a membrane-bound form of chicken ovalbumin (mOVA) as a self-antigen in the kidney and pancreas. They found that the high-affinity OVA-specific T cells caused both the rapid eradication of OVA-expressing ID8 ovarian carcinoma cells and autoimmune diabetes, in all treated mice. The low-affinity T cells, however, mediated the selective eradication of tumor cells, without any concomitant autoimmune beta cell destruction [144]. These findings were supported by the study reported by Sherman in 2008, which showed that low-affinity antigen-specific CD8 T cells tolerized with the cross-presented tumor antigen were subsequently able to eradicate tumors with the help of CD4 T cells [175]. In a therapeutic tumor vaccine study, vaccination against an antigen expressed in both tumors and normal tissues was able to induce low-avidity antigen-specific CD8+ T cells to reject tumor cells with high levels of target antigen expression, while remaining tolerant of antigen-expressing pancreatic beta cells [176]. These studies indicated that TCRs with low to medium affinities are critical components of the immune response against tumor cells. Many tumor-associated antigens are overexpressed in tumor cells with minimal or limited expression in normal tissues [20]. Moreover, studies reported that some chemicals, cytokines, and radiation therapies can activate the MHC signaling pathway and upregulate the expression of peptide/MHCs on tumor cell surfaces [177, 178], and combining immunotherapies with other therapies is the subject of active clinical investigations [179]. These indicated that TCRs with optimal low to medium affinities, when combined with other therapies, may specifically eradiate tumor cells without the induction of autoimmune diseases.

## Conclusion

Compared with the current status of CAR-T therapies in a clinic, TCR-based immunotherapies are lagging, despite their earlier inception. However, due to the unique feature of TCR-based therapies to target intracellular antigens and their significant anti-tumor effect against solid tumors, combined with the advancements in genetic engineering technologies and a growing interest from pharmaceutical companies [23], we believe that the wide application of TCR-based therapy should occur immediately and that a breakthrough of TCR-T therapies in the field of cancer immunotherapy can be predicted in the near future.

## Data Availability

The dataset supporting the conclusions of this article is included within the article.

## Abbreviations

ACT: Adoptive T cell therapy
ADCC: Antibody-dependent cell-mediated cytotoxicity
aGVHD: Autologous graft versus host disease
ALL: Acute lymphoblastic leukemia
AML: Acute myeloblastic leukemia
BITEs: Bispecific T cell engagers
CAMEL: CTL-recognized antigen on melanoma
CAR: Chimeric antigen receptor
CAR-T: Chimeric antigen receptor-engineered T cell
CD19: Cluster of differentiation 19
CD3γ: CD3 gamma chain
CD3δ: CD3 delta chain
CD3ε: CD3 epsilon
CD3ζ: CD3 zeta chain
CEA: Carcinoembryonic antigen
CR: Complete response
CRISPR: Clustered regularly interspaced short palindromic repeats
CT: Cancer testis
CTA: Cancer testis antigen
CTL: Cytotoxic T lymphocyte
DC: Dendritic cells
EBV: Epstein-Barr virus
ER: Endoplasmic reticulum
FC: Fragment crystallizable
FDA: Food and Drug Administration
FL: Fluorescein
gp100: Glycoprotein 100
GVHD: Graft versus host disease
HA-1: Minor histocompatibility antigen HA-1
HCV: Hepatitis C virus
HPV: Human papillomavirus
HSCs: Hematopoietic stem cells
IL-2: Interleukin-2
ImmTAC: Immune mobilizing monoclonal TCRs against cancer
iPSCs: Induced pluripotent stem cells
ITK: Interleukin-2 inducible tyrosine kinase
LAK: Lymphokine-activated killer
LAT: Linker for activation of T cells
LCK: Leukocyte-specific tyrosine kinase
LMP2: Latent membrane protein 2
MAGE-A1: Melanoma-associated antigen 1
MAGE-A3: Melanoma-associated antigen 3
MAPK: Mitogen-activated protein kinase
MART-1: Melanoma antigen recognized by T cells 1
MDM2: Mouse double-minute 2
MDS: Myelodysplastic syndrome
mHag: Minor histocompatibility antigens
MHC: Major histocompatibility complex
mOVA: Membrane-bound form of chicken ovalbumin
NF-κB: Nuclear factor kappa-light-chain-enhancer of activated B cells
NK: Nature killer
NS3: Non-structure protein 3
NSCLC: Non-small cell lung carcinoma
NY-ESO-1: New York esophageal squamous cell carcinoma-1
OVA: Ovalbumin
P53: Tumor protein p53
PANC-1: Pancreatic carcinoma
PBMCs: Peripheral blood mononuclear cells
PET: Positron emission tomography
PKC: Protein kinase C
Pmel-1: Premelanosome protein
R/R: Refractory or relapse
RCC: Renal cell carcinoma
RECIST: Standard criteria of response evaluation criteria in solid tumors
rhIL-2: Recombinant human IL-2
RPS4Y: Ribosomal protein S4, Y-linked
SAE: Serious adverse events
scFV: Single-chain fragment variable
scTCR: Single-chain TCR
SiRNA: Small-interfering RNAs
SLP-76: Leukocyte protein of 76 kDa
TALENs: Transcription activator-like effector nucleases
TCR: T cell receptors
TCRA: T cell receptor alpha chain
TCRB: T cell receptor beta chain
TRAV: T cell receptor alpha-chain variable
TRBV: T cell receptor beta-chain variable
T_SCM_: Memory stem cells
UTY: Ubiquitously transcribed tetratricopeptide repeat gene on the Y chromosome
VGPR: Good partial response
WT1: Wilms’ tumor 1
ZAP70: Zeta-activated protein 70 kDa
ZFNs: Zinc finger nucleases
